# RETRACTION: Mechanism of Action of Montmorillonite Powder on Injury and Repair Factors in Stage II Pressure Ulcers in Model Mice

**DOI:** 10.1111/srt.70284

**Published:** 2025-10-29

**RETRACTION**: X. Li, and J. Zhao, “Mechanism of Action of Montmorillonite Powder on Injury and Repair Factors in Stage II Pressure Ulcers in Model Mice,” *Skin Research and Technology *30, no. 8 (2024): e70010, https://doi.org/10.1111/srt.70010.

The above article, published online on 21 August 2024 in Wiley Online Library (wileyonlinelibrary.com), has been retracted by agreement between *Skin Research and Technology*; and John Wiley & Sons Ltd. The article was accepted for publication following an insufficient peer review process that does not meet the standards of the journal's peer review policy. Furthermore, scientific inconsistencies between methodology presented and reported results were identified, and the study's rationale is not adequately supported by the cited literature. Accordingly, the article has been retracted. The authors have been informed of the retraction decision but were unavailable for final confirmation.

